# Harnessing Silicon and Nanosilicon Formulations with *Rhizobium/Bradyrhizobium* for the Sustainable Enhancement of Biological Nitrogen Fixation in Legumes and Climate Change Mitigation

**DOI:** 10.3390/ijms27042031

**Published:** 2026-02-21

**Authors:** Mohamed Hemida Abd-Alla, Elhagag A. Hassan, David Mamdouh Khalaf, Esraa A. Mohammed, Shymaa R. Bashandy

**Affiliations:** Botany and Microbiology Department, Faculty of Science, Assiut University, Assiut 71516, Egypt; elhagaghassan@aun.edu.eg (E.A.H.); david.kamel@aun.edu.eg (D.M.K.);

**Keywords:** biological nitrogen fixation, *Rhizobium*/*Bradyrhizobium*, silicon nanoparticles, legumes, abiotic stress, climate-smart agriculture, greenhouse gas mitigation

## Abstract

Silicon has long been recognized as a beneficial element in plant biology. Recent advances in nanosilicon technology have revealed its transformative potential in legume-rhizobia symbiosis. This review synthesizes current knowledge on how silicon and SiO_2_ nanoparticles (Si-NPs) influence nodulation, microbial metabolism, and soil–plant interactions. We highlight emerging evidence that Si-NPs enhance symbiotic signaling, strengthen infection pathways, and mitigate oxidative stress, thereby supporting nitrogen fixation efficiency. Beyond the rhizosphere, nanosilicon improves soil structure, microbial diversity, and plant resilience under abiotic stress, offering a multifaceted approach to sustainable agriculture. The novelty of this review lies in its integrative perspective, connecting molecular mechanisms with ecological impacts and climate-smart applications. By examining Si-NPs across three domains—soils, rhizosphere metabolites, and plants—we provide a framework for understanding their role in enhancing productivity while reducing environmental costs. Importantly, we identify critical research gaps, including the need for standardized application protocols, large-scale field validation, sustainable nanosilicon production, and robust regulatory frameworks. These insights position nanosilicon as a promising tool for advancing legume productivity, reducing reliance on synthetic fertilizers, and contributing to global food security. This review underscores silicon’s potential not only as a plant nutrient but also as a strategic agent in climate-resilient agriculture.

## 1. Introduction

Legumes (*Fabaceae*) are vital for global food security, serving as a primary source of plant-based protein and providing the indispensable ecosystem service of biological nitrogen fixation (BNF) through symbiosis with rhizobia [1]. This partnership can reduce the need for synthetic nitrogen fertilizers by 20–60 kg N/ha annually [2], depending on species and environmental conditions, thereby mitigating inputs that contribute approximately 2.1–2.5% of global anthropogenic greenhouse gas emissions and drive eutrophication [3]. Instead, these technologies should be regarded as complementary strategies that decrease fertilizer requirements, improve nutrient-use efficiency, and mitigate environmental impacts, particularly when integrated into sustainable management practices. The efficiency of biological nitrogen fixation (BNF) is known to be significantly affected by climate change-induced abiotic stresses, such as drought and salinity, which can disrupt nodule formation and function [4].

Silicon (Si), the second most abundant element in Earth’s crust, is increasingly recognized as a multifunctional beneficial element that enhances plant–microbe interactions [5]. Although historically studied in Si-accumulating grasses, recent work reveals that legumes are more accurately classified as intermediate or passive Si accumulators (0.5–1.0% Si in dry weight), rather than true excluders. In contrast, most dicots, such as tomatoes and cucumbers are considered Si excluders (<0.5% Si). Even at relatively low accumulation levels, legumes can derive measurable physiological benefits from silicon application, particularly under abiotic stress conditions [6]. Silicon nanoparticles (SiO_2_ NPs) offer enhanced benefits due to their superior bioavailability and reactivity, stemming from their nanoscale size. This allows for direct cellular uptake, faster dissolution into silicic acid, and improved translocation compared to traditional silicon forms. In soybeans exposed to elevated CO_2_ levels, SiO_2_ NPs increased nodule number by approximately 48%, nodule biomass by around 54%, and nitrogenase activity by about 65% compared to plants grown under ambient conditions [7,8,9]. This review synthesizes evidence that Si and SiO_2_ NPs represent underutilized tools to safeguard and enhance the legume–rhizobia symbiosis under climate stress. We first dissect the nodulation process to pinpoint stress-vulnerable stages. We then elucidate Si-mediated protective mechanisms, and finally, we suggest integrated *Rhizobium*–nanosilicon formulations as a forward-looking strategy for climate-smart agriculture.

## 2. Molecular and Cellular Mechanisms of Nodulation

**Figure 1:**
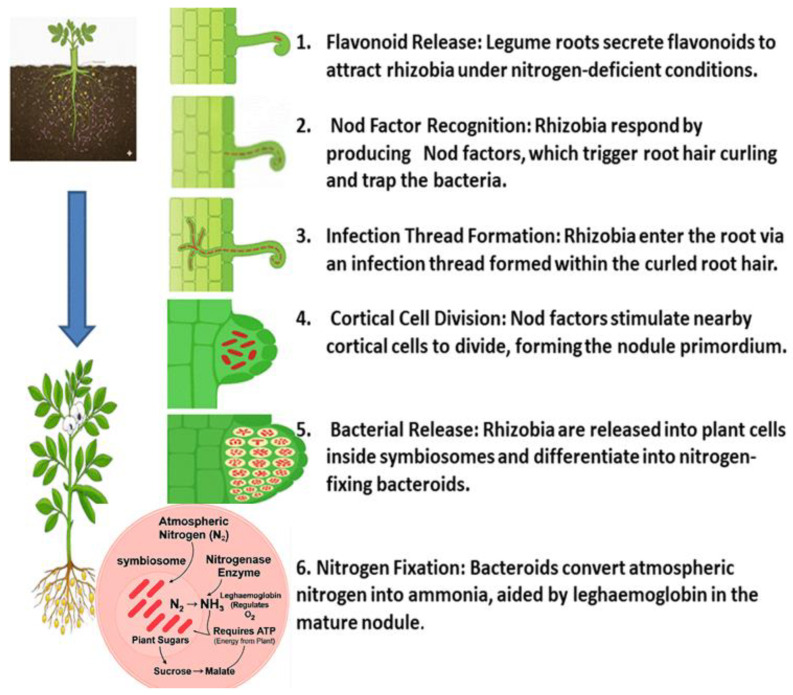
Stages of rhizobia-legume symbiosis and nitrogen fixation. This figure depicts the sequential stages of rhizobia infection and nodule formation in legumes. The process initiates with the release of flavonoids from roots in response to nitrogen deficiency, leading to the recognition of Nod factors and curling of root hairs. Subsequently, rhizobia penetrate through infection threads, inducing cortical cell division and the development of nodule primordia. Within plant cells, rhizobia transform into bacteroids within symbiosomes, ultimately converting atmospheric nitrogen into ammonia with the assistance of leghemoglobin in mature nodules.

The establishment of nitrogen-fixing root nodules in legumes is one of the most sophisticated symbiotic interactions in the plant kingdom, which demands bidirectional signal transduction between the host and its rhizobial partner. Figure 1 provides a schematic overview of the key stages, from root hair curling to mature nodule formation. The process commences when nitrogen-starved legume roots excrete flavonoids and isoflavonoids into the rhizosphere. These signal molecules are perceived by compatible rhizobia, inducing the expression of *nod* genes and the subsequent synthesis of lipochitooligosaccharide Nod factors (NFs). In the host plant, NFs are recognized by LysM receptor-like kinases, which trigger the common symbiotic signaling pathway and activate master transcriptional regulators such as NIN (Nodule Inception), thereby initiating nodule organogenesis [10].

The ensuing developmental cascade encompasses root-hair curling, entrapment of rhizobia, formation and progression of infection threads, reactivation of cortical cell divisions, and the ultimate integration of nodule vascular bundles. Each stage is governed by finely balanced hormonal and genetic networks involving cytokinin, auxin, and other phytohormones [11]. As highlighted by Ma et al. [12], nodulation is highly energy-intensive, requiring large amounts of photosynthate-derived ATP and reductant to sustain nitrogenase activity. This high metabolic demand renders the entire process acutely vulnerable to abiotic stressors such as drought, salinity, and nutrient imbalance, which disrupt signal perception, impair infection-thread progression, provoke premature senescence, and compromise nodule maintenance.

Given its pivotal role in sustainable agriculture and its inherent sensitivity to climate-driven stresses, strengthening the robustness of nodulation remains a critical strategy for reducing dependence on synthetic nitrogen fertilizers and fostering long-term soil health [13].

### 2.1. Molecular Dialog and Symbiotic Initiation

**Figure 2:**
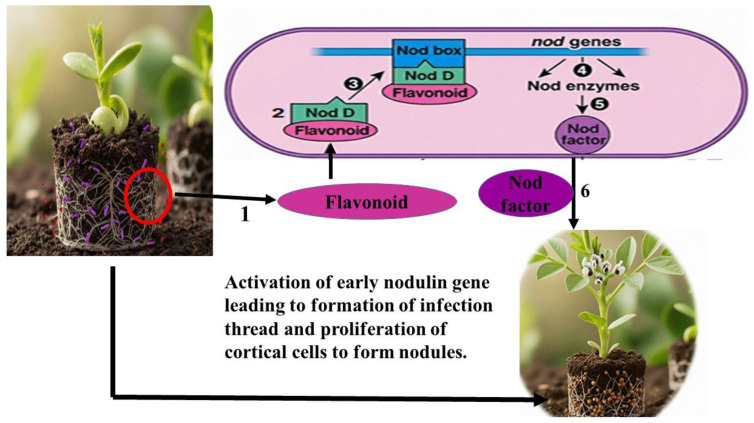
Flavonoid-induced activation of nod gene expression in legume–*Rhizobium* symbiosis. Flavonoids secreted by legume roots activate bacterial NodD proteins, which induce transcription from the nod box and expression of nod genes. The resulting Nod enzymes synthesize Nod factors that trigger host signaling cascades, leading to activation of early nodulin genes, infection thread formation, and cortical cell proliferation, culminating in root nodule development.

The establishment of the legume-rhizobia symbiosis commences with a finely tuned molecular dialog in the rhizosphere. Under nitrogen-deficient conditions, the legume host initiates this process by secreting a diverse array of flavonoid and isoflavonoid compounds. As summarized in Figure 2, these phenolic signals are perceived by compatible rhizobia, triggering a cascade that leads to successful nodulation.

Flavonoids function as highly specific chemical signals. Upon release, they are absorbed by rhizobia and recognized by the bacterial transcriptional activator protein, NodD. The binding of specific flavonoids to NodD facilitates RNA polymerase access to the nod box promoter, activating a suite of nod genes. This activation leads to the synthesis of bacterial lipochitooligosaccharide signals known as Nod Factors (NFs) [6,7,8]. The host-specificity of the symbiosis is largely determined at this stage, as only certain flavonoid-NodD pairings between a legume and a *Rhizobium* strain are effective; representative examples of these specific interactions are detailed in Table 1.

The molecular exchange continues when the plant perceives these Nod factors. This recognition is mediated by LysM domain-containing receptor-like kinases on the root hair surface (e.g., NFR1/NFR5 in *Lotus japonicus* or LYK3/NFP in *Medicago truncatula*) [19]. This perception activates the conserved common symbiotic signaling pathway (CSSP), inducing nuclear calcium spiking and the expression of early nodulin genes, which collectively prepare the plant for infection and nodule organogenesis [20]. This precise dialog is therefore essential not only for initiating symbiosis but also for ensuring the specificity required for successful nitrogen fixation [8,21].

Beyond their primary signaling role, key flavonoids are highly localized at infection sites, where they function as phytoalexins and contribute to selective symbiont recognition during the infection process [6,22,23].

### 2.2. Infection Thread Formation and Bacterial Entry

Following successful molecular recognition, the legume-rhizobia partnership advances to physical integration through a tightly regulated infection process. Nod Factor signaling induces dramatic morphological changes in root hairs, notably their curling into shepherd’s crook structures that entrap rhizobia at the site of attachment [24]. At this location, the plant cell wall undergoes invagination, forming an infection thread (IT)—which is a specialized tubular structure that facilitates bacterial entry and movement toward the root cortex [25]. The IT is lined by the plant plasma membrane and filled with a matrix, rich in arabinogalactan proteins, extensins, and pectins, that supports bacterial proliferation and directional movement [25]. The formation and elongation of the IT require precise cell wall remodeling, involving plant-derived enzymes such as cellulases and pectinases that locally modify the extracellular matrix to accommodate bacterial passage while preserving cellular integrity [26].

Recent imaging studies have revealed that infection thread (IT) formation is preceded by the development of an infection chamber, a localized, plant-derived compartment at the root hair tip where rhizobia accumulate and proliferate before entering the IT. Remodeling of this chamber, triggered by Nod factor signaling and root hair curling, initiates the inversion of the root hair cell wall that gives rise to the IT [27,28,29]. This chamber undergoes extensive remodeling, including cell wall loosening, exocytotic activity, and vesicle trafficking, processes regulated by early nodulin genes and vesicle-associated proteins [30]. Critically, host genetic factors such as NIN (Nodule Inception) orchestrate the transition from bacterial entrapment to IT initiation. However, NIN functions within a complex regulatory network involving other transcription factors (e.g., ERN, NSP1/NSP2) and signaling pathways that collectively coordinate infection thread initiation and nodule organogenesis [31]. This coordinated mechanism ultimately guides rhizobia through the root hair into cortical cells, leading to nodule formation and the establishment of nitrogen-fixing symbiosis [31].

### 2.3. Nodule Organogenesis and Bacterial Internalization

Concurrently with the advancing infection process, the legume host initiates the development of a novel organ—the root nodule—through a highly coordinated program of nodule organogenesis. This process is triggered by Nod Factor signaling, which reactivates cell division in previously quiescent cortical cells, leading to the formation of a nodule primordium [32]. The orchestration of this developmental program involves a complex interplay of phytohormones, particularly cytokinins, which activate the transcription factor Nodule Inception (NIN). NIN plays a central role in coordinating auxin redistribution and regulating genes essential for both infection and organogenesis [33,34].

As the infection thread reaches the nodule primordium, rhizobia are released into the host cytoplasm via an endocytosis-like process. In this key step, each bacterium becomes enclosed within a plant-derived membrane, forming a symbiosome [35]. Within these compartments, rhizobia differentiate into bacteroids, the nitrogen-fixing form of the bacteria. This differentiation is accompanied by extensive physiological reprogramming, including metabolic shifts and growth arrest, enabling efficient nitrogen fixation under low oxygen conditions [36].

The formation and maintenance of symbiosomes are tightly regulated by host genes, including early and late nodulin genes, which encode proteins involved in membrane trafficking, defense suppression, and nutrient exchange [32]. This intricate coordination ensures the establishment of a functional symbiotic interface capable of sustaining long-term nitrogen fixation.

### 2.4. Nodule Typology and Nitrogen Metabolism in Leguminous Plants

Leguminous plants form specialized root nodules through their mutualistic symbiosis with nitrogen-fixing rhizobia. These structures are broadly classified into two morphological types—determinate and indeterminate nodules—which differ in their development, anatomy, and metabolic characteristics [37].

Determinate nodules, characteristic of tropical legumes such as *Glycine max* (soybean) and *Phaseolus vulgaris* (common bean), originate from cortical cell divisions and exhibit a spherical morphology. A key feature is their lack of a persistent meristem, leading to early growth cessation and a relatively uniform internal structure without distinct developmental zones [38]. In contrast, indeterminate nodules, typical of temperate legumes like *Pisum sativum* (pea) and *Medicago sativa* (alfalfa), develop from both pericycle and cortical cell divisions and maintain an active apical meristem. This persistent meristematic activity produces elongated nodules with clearly defined developmental zones—including the infection, fixation, and senescence zones—reflecting a continuous gradient of symbiotic development [39].

The metabolic specialization of these nodule types extends to their nitrogen export mechanisms. Determinate nodules primarily export fixed nitrogen as ureides (allantoin and allantoic acid), which are synthesized via the purine oxidation pathway [40]. This pathway yields a transport form with a high nitrogen-to-carbon ratio, enabling efficient nitrogen transport at a reduced metabolic cost. Conversely, indeterminate nodules predominantly export amides, particularly glutamine and asparagine, synthesized through the glutamine synthetase/glutamate synthase (GS/GOGAT) pathway [41]. This divergence in nitrogen transport compounds reflects adaptations to the different ecological niches and photosynthetic capacities of their host plants.

The regulation of nodulation and nitrogen fixation involves complex signaling networks, including systemic modulation by shoot-derived isoflavonoids. As demonstrated by Abd-Alla [42] in interspecies grafts between soybean and common bean, these long-distance signals significantly influence both nodule formation and nitrogen export pathways. This regulatory mechanism ensures optimal resource allocation between nitrogen acquisition and carbon expenditure, maintaining metabolic equilibrium under varying environmental conditions [43]. Despite constituting a small fraction of total plant biomass, nodules are highly metabolically active, consuming up to 28% of the plant’s photosynthate [44]. This high cost underscores the necessity for tight regulatory control. A comprehensive understanding of nodule typology, metabolic dynamics, and systemic regulation is therefore critical for optimizing BNF to enhance both agricultural productivity and ecological sustainability.

Abiotic stresses disrupt nodulation at distinct stages. Drought most severely impairs early flavonoid signaling, infection thread progression, and nodule initiation, particularly when soil water potential falls below −0.5 to −1.0 Mpa [45]. In contrast, salinity stress (>50–100 mM NaCl) strongly disrupts infection thread integrity and nitrogenase activity, largely through ionic toxicity, altered exopolysaccharide production, auxin imbalance, and reactive oxygen species accumulation [46]. These stage-specific vulnerabilities highlight the need for targeted interventions to safeguard symbiosis under climate-driven stress. Table 2 summarizes the stage-specific vulnerabilities to drought and salinity stress in nodulation, including quantitative thresholds and molecular mechanisms. Drought mainly affects early signaling and infection thread progression, while salinity disrupts infection thread integrity and nitrogenase activity.

### 2.5. Nitrogen Fixation and Nodule Function

The functional culmination of this elaborate developmental process is nitrogen fixation itself, a biochemical process with stringent physiological requirements. Within the differentiated bacteroids, the nitrogenase enzyme complex catalyzes the ATP-dependent reduction of atmospheric dinitrogen (N_2_) to ammonia (NH_3_) [47]. A central challenge is that this remarkable enzyme is notoriously oxygen-labile. The symbiosis elegantly solves this through a coordinated mechanism: the plant produces leghemoglobin, an oxygen-binding protein that creates an essential microaerobic environment within the nodule. This system precisely regulates oxygen diffusion, simultaneously supporting bacteroid respiration while protecting nitrogenase from irreversible inactivation [48].

This sophisticated partnership operates on a principle of mutualistic exchange. The plant provides the bacteroids with carbon sources—primarily photosynthates like malate and succinate—to fuel the energy-intensive fixation process. In return, the fixed nitrogen is assimilated into organic compounds (e.g., asparagine or ureides) and exported to the plant to support growth and development [49,50]. This elegant exchange completes a remarkable cycle of biological cooperation, transforming atmospheric nitrogen into a fundamental resource for life.

## 3. Silicon in Plant Systems: From Basic Uptake to Advanced Nanotechnology

### 3.1. Silicon Availability, Uptake Mechanisms, and Accumulation Patterns

Silicon is the second most abundant element in the Earth’s crust by mass; however, its bioavailable form, monosilicic acid (H_4_SiO_4_), typically occurs in soil solutions at low concentrations ranging from 0.1 to 0.6 mM [51,52]. The biogeochemical cycling of silicon in agricultural soils is governed by factors such as pH, mineralogy, and organic matter content, with intensive cultivation often leading to significant depletion [53].

The bioavailability of silicon in soils is strongly influenced by multiple factors, including temperature (which accelerates dissolution rates), precipitation (which determines leaching versus accumulation), microbial activity (particularly phytolith dissolution), plant uptake and return through litter, and clay mineral formation that acts as a long-term sink [54]. Reported values indicate that intensive cropping can deplete plant-available silicon by 20–50% within a single growing season, while highly weathered tropical soils may contain less than 10 mg Si L^−1^ of monosilicic acid compared to >40 mg Si L^−1^ in temperate soils [55]. Such quantitative evidence supports the characterization of ‘significant depletion’ in agricultural and natural ecosystems.

Plant species vary widely in their ability to absorb and accumulate silicon and are categorized into three physiological groups—high, intermediate, and low accumulators—based on their uptake mechanisms and resultant tissue concentrations [56]. This framework is widely used, although alternative classifications have also been proposed. Active silicon accumulators such as rice and wheat (Poaceae) rely on two transporters: Lsi1, a passive aquaporin channel mediating silicon influx, and Lsi2, an active efflux transporter responsible for xylem loading [54]. Their coordinated activity enables grasses to reach up to 10% silicon in shoot dry weight [57,58]. Intermediate accumulators like cucumbers and melons achieve moderate levels (1–3%) through combined passive and active uptake strategies [59]. In contrast, most legumes, including soybean and common bean, lack functional homologs of Lsi1/Lsi2, reflecting evolutionary divergence in root anatomy and transporter gene families. Consequently, they are classified as silicon excluders, typically accumulating less than 0.5% in shoot tissues [60,61,62]. Transporter expression is tightly regulated by silicon availability, with evidence of silicon-responsive cis-elements and transcription factors such as MYB and NAC modulating activity [62].

Although legumes are generally considered excluders, recent findings suggest their role has been underestimated. Even at low tissue concentrations (<0.5% dry weight), silicon enhances resistance to fungal pathogens and improves tolerance to drought and salinity stress [53,63]. The absence of specialized influx and efflux transporters limits accumulation, yet physiological benefits remain significant [54,62]. As emphasized by Katz [64], tissue concentration alone does not fully capture silicon’s functional impact. Critically, even low levels of silicon accumulation can impart significant physiological advantages, particularly under abiotic and biotic stress. This evolving understanding is especially pertinent to legumes, where silicon-mediated benefits—such as enhanced cellular integrity, improved photosynthetic efficiency, and strengthened symbiotic interactions—may arise through mechanisms independent of high tissue accumulation [65].

### 3.2. The Nanosilicon Revolution: Enhanced Delivery and Efficacy

The development of silicon nanoparticles (SiO_2_ NPs, 1–100 nm) marks a significant advancement in plant nutrition and stress mitigation, offering distinct advantages over conventional silicon fertilizers [66,67]. Their nanoscale dimensions impart unique physicochemical properties such as a high surface area-to-volume ratio, size-dependent quantum confinement effects that alter electronic band structures and optical absorption, and elevated surface reactivity that enhance interactions with biological systems [68].

**Figure 3:**
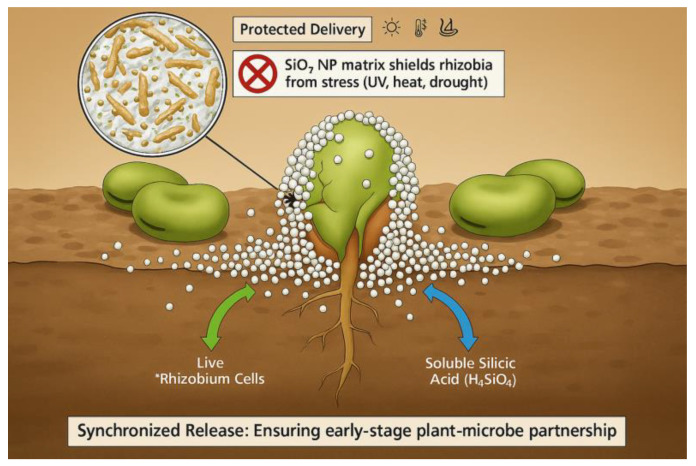
Nanosilicon matrix-mediated protection and synchronized delivery in *Rhizobium*–plant symbiosis. The SiO_2_ nanoparticle matrix encapsulates live rhizobial cells, shielding them from environmental stressors such as UV light, heat, and drought, while simultaneously releasing soluble silicic acid (H_4_SiO_4_). This synchronized delivery ensures early establishment of the plant–microbe partnership and sustained stress tolerance benefits into the root zone ensures optimal availability of both the microbial inoculant and the biostimulant during the critical phase of early legume–rhizobia symbiosis.

A key advantage of nanosilicon is its superior bioavailability. Studies across diverse species—including rice, wheat, maize, cucumber, and soybean—have shown that SiO_2_ nanoparticles are absorbed more efficiently by plant roots and foliage than traditional silicon sources, thereby circumventing the transporter limitations that restrict silicon uptake in legume species [66,69,70]. This enhanced uptake is particularly valuable for improving silicon-mediated responses in crops typically classified as silicon excluders. In addition, nanosilicon formulations exhibit controlled-release behavior, gradually solubilizing to maintain sustained levels of plant-available silicic acid in the rhizosphere [67]. Mechanistic evidence indicates that SiO_2_ NPs enter plants through multiple pathways: partial dissolution to monosilicic acid enabling transport via Lsi1 aquaporin channels, direct endocytosis of nanoparticulate forms, and enhanced root surface adsorption [66,70]. Reported efficiency gains are species- and dose-dependent, with 2–5 fold higher silicon accumulation in shoots of rice and wheat treated with SiO_2_ NPs compared to equivalent concentrations of bulk SiO_2_ [69,70]. Collectively, these findings support the conclusion that SiO_2_ NPs are absorbed more efficiently, both in terms of uptake rate and tissue accumulation, than conventional silicon sources. As illustrated in Figure 3, the proposed mechanism involves the synchronized delivery and rhizosphere interaction of a nano-biofertilizer. This granular composite incorporates *Rhizobium* cells immobilized within a porous SiO_2_ nanoparticle (NP) matrix. The engineered matrix enhances bacterial resilience by shielding the encapsulated cells from environmental stressors such as ultraviolet radiation, temperature extremes, salinity, and desiccation. Simultaneously, the controlled release of viable *Rhizobium* cells and soluble silicic acid (H_4_SiO_4_).

The SiO_2_ NP matrix thus performs a dual function: (i) protection of live *Rhizobium* cells against abiotic stressors and (ii) gradual dissolution to sustain bioavailable silicon in the rhizosphere. This synchronized delivery promotes a resilient plant–microbe partnership, strengthening nodulation efficiency, root system development, and stress tolerance during the early growth stages.

**Figure 4:**
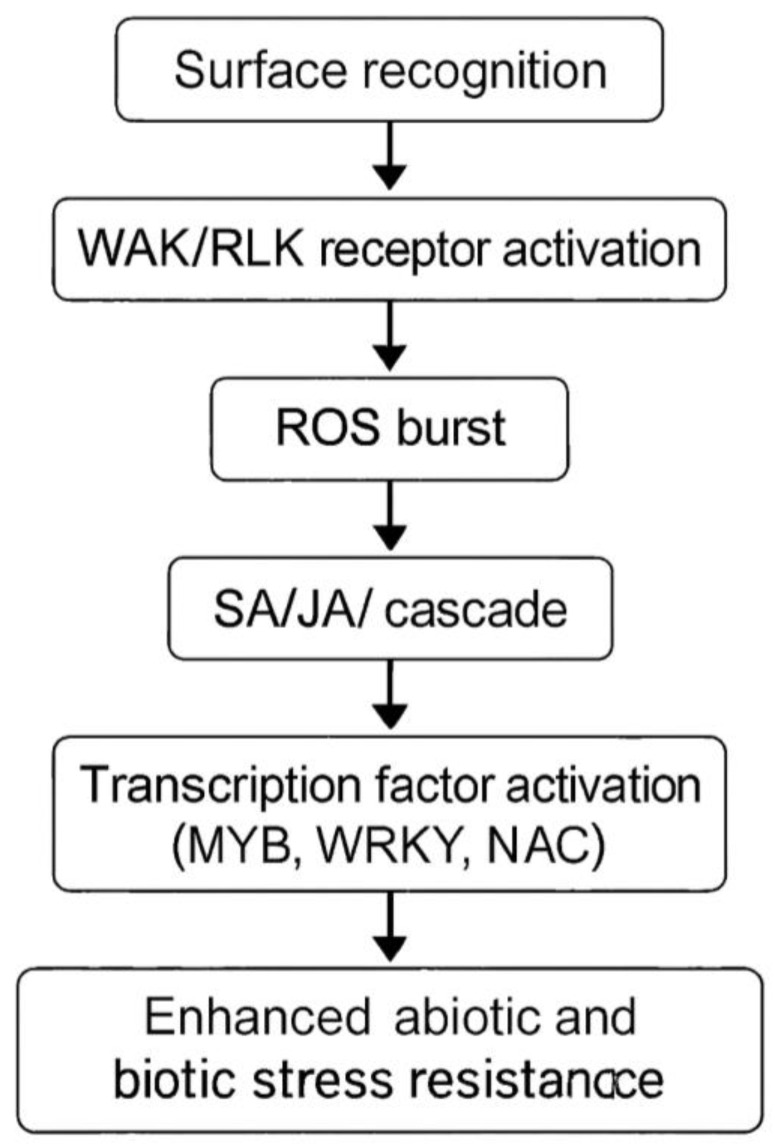
Nanosilicon recognition and early defense signaling in plant cells. SiO_2_ nanoparticles and dissolved silicic acid (H_4_SiO_4_) interact with wall-associated kinases (WAKs), receptor-like kinases (RLKs), and aquaporin channels (Lsi1). These recognition events initiate apoplastic perturbations that activate early defense signaling pathways.

Notably, nanosilicon exhibits a superior capacity to elicit plant defense responses compared to conventional bulk silicon sources. The unique surface characteristics of SiO_2_ nanoparticles—including charge distribution, nanoscale size, and dissolution behavior—enhance their interactions with cell wall–associated receptors. Wall-associated kinases (WAKs) and receptor-like kinases (RLKs) perceive apoplastic perturbations caused by nanoparticle adsorption or partial dissolution, thereby activating downstream defense signaling pathways (Figure 4) [70,71].

**Figure 5:**
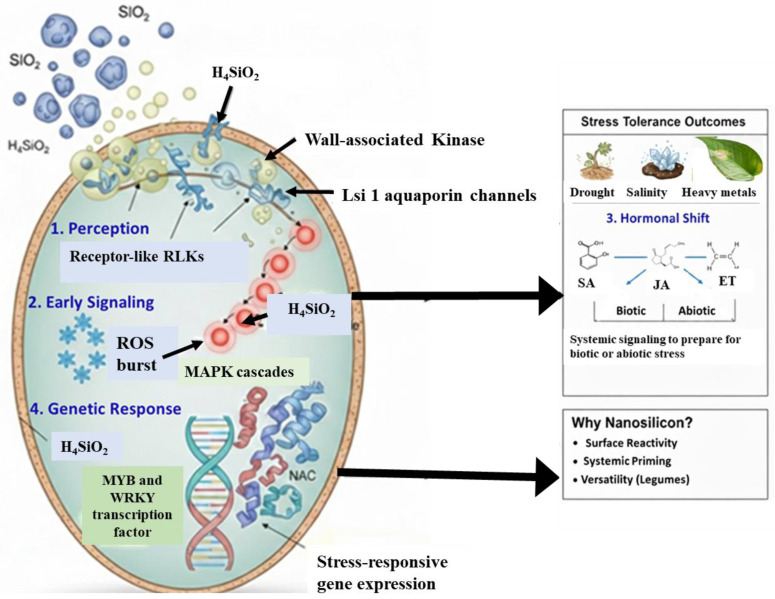
Nanosilicon-induced defense signaling cascade in plants. Perception of nanosilicon triggers reactive oxygen species (ROS) bursts and MAPK cascade activation, leading to modulation of salicylic acid (SA), jasmonic acid (JA), abscisic acid (ABA), and ethylene (ET) pathways. Transcription factors, including MYB, WRKY, and NAC families, are subsequently upregulated, coordinating stress-responsive gene expression and conferring enhanced tolerance to abiotic and biotic stresses.

Dissolved silicic acid (H_4_SiO_4_) may also enter through Lsi1 aquaporin channels, indirectly influencing signaling. These recognition events initiate defense cascades, including reactive oxygen species (ROS) bursts, mitogen-activated protein kinase (MAPK) activation, and downstream modulation of salicylic acid (SA), jasmonic acid (JA), and ethylene (ET) pathways [70] as shown in Figure 5. Transcription factors such as MYB, WRKY, and NAC families are subsequently upregulated, coordinating stress-responsive gene expression [53]. The high reactivity and distinctive surface properties of SiO_2_ nanoparticles further enable effective activation of systemic acquired resistance and antioxidant defense pathways compared to conventional silicon formulations [67,72]. This enhanced priming effect contributes to improved tolerance against diverse abiotic stresses—including drought, salinity, and heavy metal toxicity—as well as biotic pressures from pathogens and insect herbivores [66,73]. Collectively, these multifaceted benefits position nanosilicon as a promising strategy for sustainable crop enhancement, particularly in legume–rhizobia symbiotic systems where traditional silicon fertilization has shown limited efficacy due to inherent uptake constraints.

## 4. Silicon-Mediated Resilience of the Legume–*Rhizobium* Symbiosis: Mechanisms of Action

**Figure 6:**
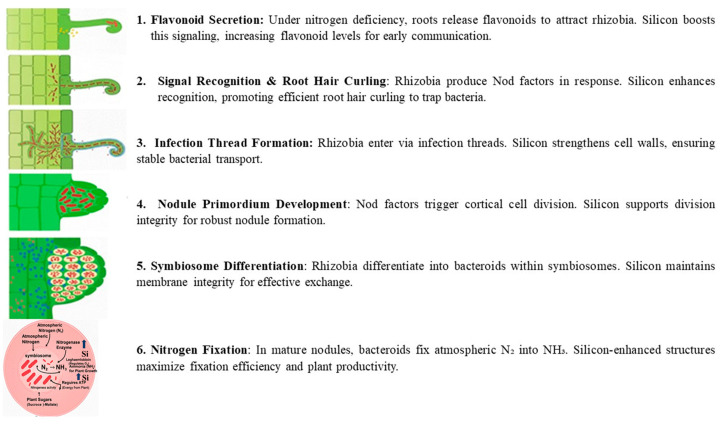
Silicon-mediated enhancement of rhizobia–legume symbiosis. This figure illustrates how silicon strengthens multiple stages of the rhizobia–legume symbiotic relationship. Silicon boosts flavonoid secretion for early signaling, enhances Nod factor recognition and root hair curling, stabilizes infection thread formation, supports cortical cell division, and maintains symbiosome integrity. In mature nodules, silicon-enhanced structures maximize nitrogen fixation efficiency, thereby improving plant productivity and resilience under stress conditions.

The processes of nodulation and biological nitrogen fixation (BNF) in legumes are highly sensitive to environmental stress and involve several well-defined physiological bottlenecks. These include the irreversible inactivation of nitrogenase by oxygen, necessitating a tightly regulated microaerobic environment that prevents oxygen from reaching and destroying the enzyme; the high energy demand of nitrogen reduction, which consumes large amounts of ATP and reducing equivalents; and carbon supply limitations, particularly during early symbiosis when photosynthetic output is insufficient to sustain bacteroid metabolism [12,74,75]. Silicon and silicon nanoparticles (SiO_2_ NPs) have emerged as promising agents to mitigate these constraints and enhance symbiotic performance, especially under abiotic stress conditions. Their beneficial effects operate through three interconnected mechanisms that collectively improve nodulation efficiency, stress tolerance, and nitrogen fixation [76], as illustrated in Figure 6. First, silicon enhances molecular signaling by stimulating root flavonoid exudation, which attracts rhizobia and promotes Nod factor synthesis [77]. Second, it reinforces the infection process by depositing silica along infection thread walls, improving structural integrity and facilitating bacterial entry [9]. Finally, within the nodule, silicon boosts antioxidant enzyme activity—superoxide dismutase (SOD), catalase (CAT), and ascorbate peroxidase (APX)—to neutralize reactive oxygen species (ROS), while stabilizing leghemoglobin to protect the oxygen-sensitive nitrogenase enzyme [76]. These combined actions create a favorable microenvironment for efficient nitrogen fixation, enabling bacteroids to convert atmospheric nitrogen (N_2_) into ammonia (NH_3_) in exchange for plant-derived carbon.

### 4.1. Enhancing Symbiotic Signaling and Nodulation

Silicon plays a crucial role in optimizing early molecular communication between legumes and rhizobia. Silicon application enhances root hair development and stimulates the synthesis and exudation of key flavonoids, including daidzein and genistein, in soybean [65]. Flavonoids are essential secondary metabolites in legumes, serving as defense compounds and signaling molecules in rhizobia-legume symbiosis. Seventeen flavonoid compounds have been identified from nodule tissues, including eight isoflavones and nine non-isoflavones. Isoflavones such as daidzein, formononetin, glycitein, and medicarpin are particularly important as root exudates that activate rhizobial *nod* genes (e.g., *nodD*), thereby initiating symbiotic signaling [78].

Treatment with SiO_2_ nanoparticles upregulated flavonoid biosynthesis genes (e.g., chalcone synthase, CHS) and increased the accumulation of isoflavones, providing molecular evidence that silicon enhances the transcriptional activity of flavonoid pathways and boosts the production of critical signaling molecules for nodulation. Elevated flavonoid levels under SiO_2_ NP treatment may also enhance stress tolerance by strengthening antioxidant defenses [78]. These compounds serve as molecular signals that activate rhizobial nod genes and initiate the symbiotic cascade. The improved flavonoid profile enhances partner recognition and facilitates infection thread formation, leading to increased nodule number and biomass. For example, under elevated CO_2_ conditions, nano-SiO_2_ application resulted in a 48.3% increase in nodule number and a 53.6% increase in nodule biomass in soybean [9].

Moreover, silicon has been reported to enhance nodulation and nitrogen fixation in legumes. Since ENOD40 and NIN (Nodule Inception) are key regulators of nodule organogenesis, it is plausible that silicon may influence their expression, although direct molecular evidence is still limited [79]. Beyond symbiosis, silicon regulates gene expression associated with stress tolerance. Recent studies in soybean (*Glycine max*) have shown that silicon supplementation, especially when combined with S-nitroso glutathione (GSNO), enhances salt tolerance by modulating key stress-responsive genes. Specifically, silicon treatment upregulated GmNHX1, GmSOS2, and GmAKT1, involved in ion transport and homeostasis, while downregulating aquaporin genes (GmNIP2.1, GmNIP2.2) and lipid biosynthesis-related GmLBR. These transcriptional changes were accompanied by improved physiological traits such as increased plant height, chlorophyll content, nodule number, and reduced oxidative damage markers, highlighting silicon’s dual role in metabolic regulation and stress resilience. These findings underscore silicon’s ability to strengthen both metabolic and signaling pathways, enhancing resilience to salinity stress and supporting sustainable legume productivity [80].

### 4.2. Ameliorating Oxidative and Nitrosative Stress

**Figure 7:**
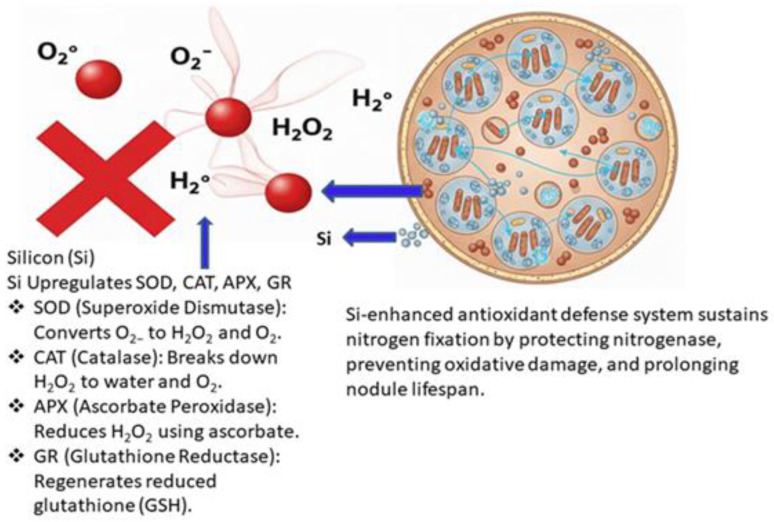
Silicon-enhanced antioxidant defense and nitrogen fixation. Silicon (Si) supplementation strengthens the antioxidant defense system in legume root nodules by upregulating key enzymes, including superoxide dismutase (SOD), catalase (CAT), ascorbate peroxidase (APX), and glutathione reductase (GR). These enzymes collectively neutralize reactive oxygen species (ROS) such as superoxide (O_2_•^−^) and hydrogen peroxide (H_2_O_2_), thereby protecting nitrogenase activity, preventing oxidative damage, and prolonging nodule lifespan. Through this enhanced defense, Si sustains efficient nitrogen fixation under stress conditions.

Biological nitrogen fixation is highly susceptible to oxidative damage due to the metabolic intensity and oxygen sensitivity of nitrogenase. Silicon counteracts this by enhancing the plant’s antioxidant defense system, upregulating enzymes such as SOD, CAT, APX, and glutathione reductase (GR) [81]. Notably, SiO_2_ nanoparticles elicit a stronger and faster antioxidant response than bulk silicon sources, as depicted in Figure 7. In drought-stressed soybean, SiO_2_ NP treatment increased antioxidant enzyme activities by 15–22% and reduced oxidative stress markers—malondialdehyde (MDA) and hydrogen peroxide (H_2_O_2_)—by 18–22% [82,83].

#### 4.2.1. Effect of Silicon on Leghemoglobin (Lb) Gene Expression

**Figure 8:**
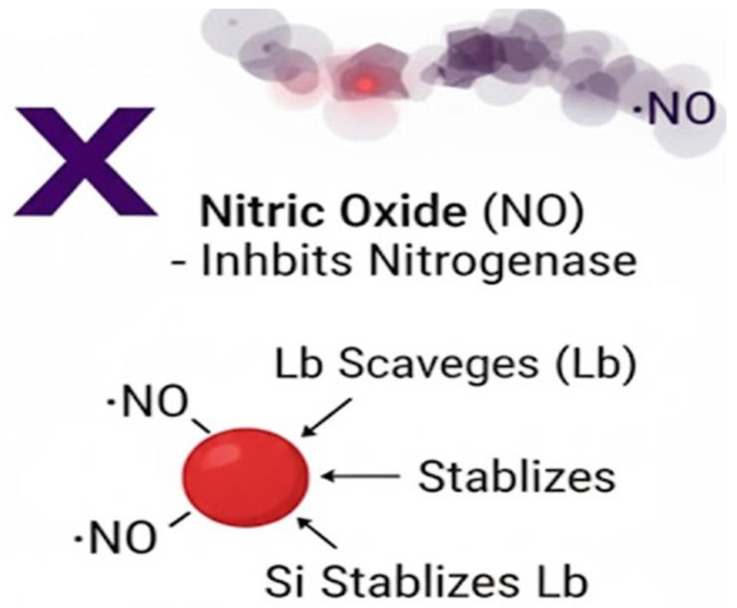
Silicon-enhanced nitrosative stress defense in nodules. Nitric oxide (NO) can inhibit nitrogenase activity in legume root nodules, compromising symbiotic nitrogen fixation. Leghemoglobin (Lb) scavenges •NO to mitigate this inhibition, and silicon (Si) further stabilizes Lb, enhancing its protective function. Through this stabilization, Si reduces nitrosative stress, preserves nitrogenase activity, and sustains efficient nodule function under adverse conditions.

Leghemoglobin (Lb) is a critical oxygen-buffering protein in legume nodules, encoded by multiple isoforms such as *LbA*, *LbB*, and *LbC*. Its stability is essential for maintaining the microaerobic environment required for nitrogenase activity. Under stress conditions, Lb is highly vulnerable to oxidative damage from reactive oxygen species (ROS), nitrosative stress from nitric oxide (•NO), and protease activity during nodule senescence (Figure 8). Silicon supplementation has been shown to mitigate these threats by enhancing antioxidant defenses, reducing ROS accumulation, and stabilizing Lb against degradation. El Moukhtari et al. [77] demonstrated that silicon treatments increase Lb levels in salinity-stressed legumes, while Tong [9] reported that nano-SiO_2_ improves soybean physiology under elevated CO_2_, linking directly to enhanced Lb formation.

#### 4.2.2. Influence on Lb Gene Expression and Synthesis

Beyond protein stabilization, silicon also influences Lb synthesis at the transcriptional level. Studies indicate that silicon, often in synergy with nitric oxide (NO), upregulates the expression of *LbA* and *LbB* genes, as well as ferric leghemoglobin reductase (FLbR) genes, which are crucial for maintaining Lb in its functional ferrous form (Lb^2+^). Lee et al. [84] reported that silicon and NO donors such as GSNO stimulate Lb synthesis and gene expression, thereby supporting O_2_ homeostasis in nodules. While direct evidence for *LbC* regulation remains limited, transcriptomic analyses suggest that silicon broadly enhances Lb isoform expression under stress. In addition, silicon supplementation improves nodulation efficiency and sustains symbiotic gene processes, indirectly supporting Lb synthesis.

#### 4.2.3. Protein Turnover and Stability

The protective effect of silicon is most pronounced at the level of protein turnover. By boosting antioxidant enzyme activity—such as superoxide dismutase (SOD), catalase (CAT), and ascorbate peroxidase (APX)—silicon reduces ROS accumulation and delays nodule senescence. This indirectly lowers protease activity, thereby reducing Lb degradation. Tong [9] highlighted that nano-SiO_2_ enhances antioxidant capacity in soybean nodules, while Lamsaadi et al. [85] showed that silicon supplementation maintains Lb content and nitrogenase activity in fenugreek under salinity stress. These findings confirm that silicon’s primary role is to stabilize existing Lb pools, ensuring prolonged nitrogenase activity.

#### 4.2.4. Balance Between Synthesis and Turnover

Taken together, silicon exerts a dual effect on Lb: it protects against protein turnover through ROS mitigation and protease inhibition, while also enhancing synthesis via upregulation of *LbA* and *LbB* gene expression. This balance sustains Lb concentrations in nodules under stress conditions such as salinity, heavy metals, and elevated CO_2_. Kaur et al. [86] and Zhou et al. [87] reported that untreated nodules under these stresses show rapid Lb decline and senescence, whereas silicon supplementation maintains nodule health and symbiotic efficiency. Thus, silicon acts both as a stabilizer of existing Lb proteins and as a regulator of gene expression, collectively ensuring efficient nitrogen fixation.

### 4.3. Providing Structural and Metabolic Support

Silicon contributes to physical reinforcement through its polymerization as amorphous silica (SiO_2_·nH_2_O) in cell walls and apoplastic spaces. This deposition strengthens root hairs and infection threads, improving resistance to mechanical stresses such as soil compaction and drought-induced shrinkage [53]. The resulting silica-phytolith layer also reduces cuticular water loss and acts as a barrier against pathogens.

Metabolically, silicon enhances photosynthetic efficiency by protecting chloroplast ultrastructure, promoting chlorophyll synthesis, and regulating stomatal function under stress [67]. These improvements ensure a steady supply of carbon skeletons and ATP—a critical consideration given that nitrogen fixation requires 16 ATP per N_2_ molecule reduced [88]. Furthermore, silicon improves water-use efficiency by modulating stomatal conductance and osmotic balance, thereby maintaining turgor pressure essential for nutrient transport and nodule function [89].

**Figure 9:**
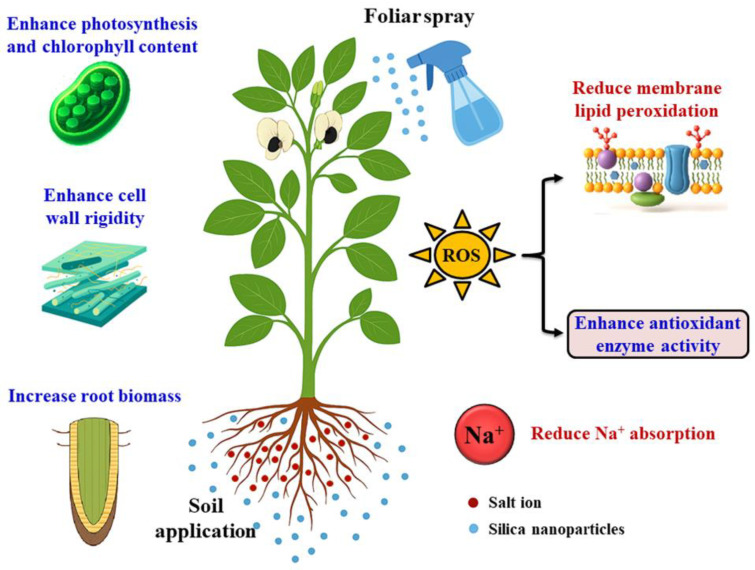
The effects of silicon nanoparticles on plant physiology and stress conditions. Silicon nanoparticles (Si-NPs) applied through foliar spray or soil amendment enhance multiple physiological processes and stress responses in plants. Si-NPs improve photosynthesis and chlorophyll content, strengthen cell wall rigidity, and increase root biomass. They also reduce reactive oxygen species (ROS) damage by enhancing antioxidant enzyme activity and limiting membrane lipid peroxidation. In saline environments, Si-NPs decrease sodium (Na^+^) uptake, thereby mitigating salt stress. Collectively, these effects contribute to improved plant growth, resilience, and productivity under adverse conditions.

The multifaceted physiological effects of silicon nanoparticles (Si-NPs) are summarized in Figure 9. The application of Si-NPs enhances photosynthetic efficiency, chlorophyll content, cell wall strength, antioxidant enzyme activity, and root biomass, while concurrently reducing Na^+^ absorption and membrane lipid peroxidation. These changes collectively enhance plant growth and development under stress conditions, particularly salinity [67].

The boosted antioxidant enzyme activity upon Si-NP application helps neutralize reactive oxygen species (ROS) and reduce oxidative damage, thereby minimizing membrane lipid peroxidation and maintaining cellular integrity [90]. Studies confirm that Si-NPs enhance photosynthetic pigments, nitrogen metabolism, and overall plant biomass under stress [67,91]. Additionally, Si-NPs improve root biomass and architecture, enhancing water and nutrient uptake, while their ability to reduce Na^+^ absorption mitigates ionic imbalance under salinity stress [92]. The strengthening of cell walls via silica deposition provides a physical barrier against both abiotic stress and pathogens [93]. Finally, soil application of Si-NPs promotes a sustained release of silicon and positive interaction with rhizospheric microbes, thereby improving nutrient cycling and overall plant resilience [94].

### 4.4. Silicon Interactions with Macronutrients

The interaction between silicon (Si) and key nutrients such as phosphorus (P) and potassium (K) in the rhizosphere plays a significant role in modulating legume nodulation and biological nitrogen fixation (BNF) efficiency. Legumes require a significant amount of phosphorus (P) for effective symbiosis. Nodules serve as strong sinks for this nutrient to support the energy-intensive activity of nitrogenase and sustain the proliferation of rhizobia. Low P availability often suppresses nodulation and BNF by limiting ATP production and carbon allocation to roots. Si supplementation can indirectly influence these processes by altering rhizosphere chemistry and nutrient dynamics [95]. Si has been shown to enhance overall plant growth and nodulation under various conditions, alleviating nutrient limitations that otherwise constrain BNF. In lucerne (*Medicago sativa*), Si addition promoted root nodulation by approximately 50% and plant growth by 93% across CO_2_ and temperature regimes [96].

Although direct studies on Si-P antagonism or synergy in legume nodules are limited compared to monocots, evidence indicates that Si can interact with P availability to support nodulation. In P-limited systems, legumes prioritize nodule P homeostasis to sustain BNF, and Si may contribute by improving root architecture or microbial activity in the rhizosphere, which enhances P mobilization. Reviews highlight that microbial phosphate solubilization stimulates BNF in grain legumes, and Si’s role in promoting beneficial rhizosphere microbes could amplify this effect. Si supplementation has restored nodulation levels comparable to those under optimal conditions in climate-stressed scenarios, where P limitation often exacerbates declines in BNF. This positions Si as a modulator that could mitigate P deficiencies’ negative impacts on symbiotic N_2_ fixation [97,98].

Potassium (K) interactions with Si in the rhizosphere are less extensively documented for legumes but appear complementary in supporting nodulation and BNF. K is essential for enzyme activation, osmotic regulation, and nodule function, while Si often enhances K uptake in plants under stress. In salinity-stressed legumes, combined Si and beneficial non-rhizobial bacteria (which can influence nutrient cycling, including K) more effectively improved nodulation and nitrogen fixation than single applications. Si may facilitate better K nutrition in nodules, indirectly boosting nitrogenase activity and symbiotic performance. Field-relevant observations suggest that Si-enriched conditions promote rhizodeposition of N compounds, linked to enhanced nodulation and influenced by balanced macronutrient availability, including K [99].

Overall, Si’s positive influence on legume-rhizobia symbiosis often occurs in nutrient-constrained environments, where interactions with P and K help sustain high BNF rates. By promoting nodule number, size, and nitrogenase content—sometimes under low nutrient or stress conditions—Si enables legumes to maintain efficient N acquisition without excessive reliance on synthetic fertilizers. For example, in *Trifolium incarnatum*, Si supply increased nodule nitrogenase and N_2_ fixation capacity, leading to greater rhizodeposited N transfer in associations, effects amplified by optimized P and K status in the rhizosphere [100,101].

These interactions underscore Si’s potential as a sustainable enhancer of legume productivity, particularly in soils with suboptimal P or K. While much research focuses on Si’s direct effects on nodulation, emerging evidence points to synergistic roles with macronutrients in regulating rhizosphere processes critical for BNF. Further targeted studies on Si-P-K dynamics in diverse legume species and field settings could clarify mechanisms and optimize applications for improved symbiotic efficiency and reduced fertilizer dependency [65,76]. Table 3 summarizes the physiological and biochemical enhancements conferred by silicon and nanosilicon. Data from recent studies show marked increases in nodule formation and biomass in soybean, likely driven by improved flavonoid signaling. Concurrently, key nitrogen-assimilation enzymes are upregulated, and antioxidant defenses are strengthened, supporting sustained nitrogen fixation under adverse conditions.

The physicochemical properties of nanosilicon confer distinct advantages over conventional silicon fertilizers, as systematically compared in Table 4. Bulk silicates must first undergo solubilization into monosilicic acid [Si(OH)_4_] to become bioavailable, a process that results in low uptake efficiency and necessitates high application rates [51]. In contrast, nanosilicon exhibits enhanced solubility and reactivity due to its high surface area, enabling effective uptake at significantly lower doses [69,106].

This advantage is particularly critical for crops such as soybean, which lack efficient silicon transporters and therefore accumulate only minimal amounts of silicon from conventional sources [107]. Nanosilicon offers a promising alternative by bypassing these physiological constraints, entering root tissues via apoplastic pathways or endocytosis, and thereby enhancing silicon nutrition in non-accumulator species [108].

**Table 4:** Comparison of conventional silicon and nanosilicon (SiO_2_ nps) for agricultural use.

Attribute	Conventional Si	Nanosilicon (SiO_2_ NPs)
Bioavailability	Low; requires solubilization into silicic acid (Si(OH)_4_) for plant uptake [109].	High; nanoparticles are readily available and can be absorbed directly, bypassing transporter limitations [69].
Application Efficiency	High application rates (e.g., several tons/hectare) are often needed to be effective due to low solubility [109].	Effective at significantly lower doses due to high surface area and reactivity [106].
Uptake Mechanism	Limited; species like soybeans lack efficient silicon transporters, leading to poor accumulation [107].	Potential for enhanced/alternative uptake via apoplastic pathways or endocytosis, bypassing transporter limitations [108].
Potential Risks and Limitations	High application rates may be cost-prohibitive and energetically inefficient. Efficacy is limited in non-accumulator species. Production costs remain high, and the regulatory framework is underdeveloped [60].	Potential ecotoxicity at high doses; unknown long-term soil fate; high production costs; underdeveloped regulatory framework.

While SiO_2_ nanoparticles attract considerable attention for their agricultural benefits—including improved nutrient uptake, enhanced plant growth, and contributions to environmental remediation—their potential ecotoxicological risks require careful evaluation. Evidence shows that SiO_2_ NP exposure can significantly disrupt soil microbial communities, which are essential for maintaining soil fertility and ecosystem stability [94]. For example, foliar application of SiO_2_ NPs at 5 mg/L (particle size ~20–50 nm) every three days for 15 days altered soil metabolite profiles and microbial community composition in *Brassica chinensis* rhizospheres [110]. Similarly, soils treated with SiO_2_ NPs in the 10–100 mg/kg range exhibited reduced microbial biomass and diversity, impairing key processes such as organic matter decomposition and nutrient cycling [111]. These disruptions cascade through soil ecosystems, ultimately compromising plant growth and resilience. SiO_2_ NPs may also affect soil biota, including plants, invertebrates, and microorganisms, as well as enzymatic activity, emphasizing the need for biomarkers to monitor these impacts [112,113].

Beyond soil systems, the accumulation of SiO_2_ NPs in plants raises concerns for food safety and human health. SiO_2_ NPs can be absorbed and translocated to edible tissues, where they may reach levels that pose risks to consumers [114,115,116]. Although such nanoparticles may enhance plant tolerance to abiotic stressors, their persistence in the food chain could have unforeseen consequences, particularly with chronic exposure. This highlights the importance of long-term studies assessing nanoparticle accumulation in relation to both food safety and ecosystem integrity.

Additionally, agricultural runoff introduces SiO_2_ NPs into aquatic ecosystems, creating further risks. These particles can leach into waterways, disrupt aquatic organisms, and destabilize food webs [117]. Bioaccumulation in aquatic species may then propagate through trophic levels, threatening biodiversity and food security. Taken together, these findings underscore the need for a balanced approach that weighs the agronomic advantages of SiO_2_ NPs against their ecological and health risks. Sustainable agricultural practices must therefore prioritize long-term soil health, ecosystem integrity, and food safety.

## 5. Nano-Biofertilizers: The Next Frontier

### 5.1. The Concept of Nano-Biofertilizers

The convergence of nanotechnology and microbial inoculants represents a transformative advancement in biofertilizer development. Co-formulating *Rhizobium or Bradyrhizobium* strains with nanomaterial carriers such as silicon nanoparticles (SiO_2_ NPs), chitosan, polymeric matrices, or other metal oxide and carbon-based systems has led to the emergence of a novel class of nano-biofertilizers. These formulations exploit the unique physicochemical properties of diverse nanomaterials to synergistically enhance microbial functionality and plant performance. This approach addresses key limitations of conventional biofertilizers, such as poor shelf life, inconsistent field efficacy, and limited stress tolerance, while introducing new capabilities that significantly improve symbiotic nitrogen fixation in legume cropping systems. The development of these advanced formulations marks a critical milestone in precision agriculture, enabling targeted delivery and improved efficacy of both microbial and mineral components (Table 5).

Traditional biofertilizer formulations often face challenges such as poor shelf life, inconsistent field performance, and limited stress resilience. By integrating *Rhizobium* or *Bradyrhizobium* strains with nanomaterial carriers like SiO_2_ nanoparticles, chitosan, polymeric matrices, or other advanced platforms, researchers have demonstrated enhanced microbial survival, root colonization, and symbiotic nitrogen fixation in legumes [65,80,118,119]. These nano-enabled formulations leverage the unique properties of nanomaterials to offer controlled release, protection against abiotic stress, and targeted delivery of beneficial microbes, marking a significant advancement in precision agriculture. The term “co-formulating” refers to the incorporation of microbial inoculants with nanocarriers through various mechanisms, including encapsulation within polymeric or chitosan matrices, surface adsorption onto nanoparticles (e.g., SiO_2_ NPs), or co-delivery systems that combine microbes and nanomaterials in a single formulation. Each of these strategies contributes to enhancing microbial persistence and functional performance in the rhizosphere.

Chitosan nanoparticles (CNPs) have emerged as particularly promising carriers for rhizobial delivery due to their biocompatibility, biodegradability, and antimicrobial properties. Encapsulation of plant growth-promoting rhizobacteria (PGPRs) in chitosan–alginate nanocapsules has been shown to improve microbial viability, water retention, and controlled release of inoculants, thereby enhancing nodulation and plant growth under stress conditions [120,121,122,123]. Moreover, chitosan nanoparticles can suppress soil-borne pathogens, indirectly supporting rhizobial colonization and symbiotic efficiency. Their dual role as protective carriers and bioactive elicitors positions CNPs as sustainable alternatives to mineral-based carriers in biofertilizer formulations.

Polymeric nanoparticles (PNPs), including poly(lactic co glycolic acid) (PLGA) and polycaprolactone (PCL), offer another versatile platform for microbial delivery [124]. These biodegradable polymers allow tunable degradation rates, enabling precise control over microbial release at critical stages of root colonization [125]. Recent studies have shown that polymeric nano formulations can encapsulate both microbial inoculants and signaling molecules, such as flavonoids, thereby enhancing rhizobial recognition and nodulation efficiency [126,127,128,129]. Polymeric nanoparticles also improve microbial stability and bioavailability, making them attractive candidates for large-scale application in legume cropping systems [130,131,132,133,134].

Collectively, SiO_2_, chitosan, and polymeric nanoparticles represent the next frontier in nano-biofertilizer development. Each carrier offers unique advantages: SiO_2_ enhances flavonoid exudation and stress resilience; chitosan provides biocompatibility and pathogen suppression; and polymeric nanoparticles enable sustained microbial release and co-delivery of signaling molecules. While challenges remain regarding scalability, production costs, and long-term ecological impacts, these nano-carriers provide a powerful toolkit for enhancing symbiotic nitrogen fixation and advancing sustainable agriculture (Table 5).

### 5.2. Enhanced Microbial Protection and Viability

Traditional rhizobial biofertilizers often suffer from short shelf life and reduced field performance due to microbial sensitivity to abiotic stressors like desiccation, UV radiation, temperature fluctuations, and soil salinity [135,136]. Nano-carrier delivery systems, engineered from materials such as silica, chitosan, or polymers, offer a promising solution by encapsulating and shielding microbial cells from these adverse conditions.

The principle of enhancing microbial resilience through protective additives is well-illustrated by Elsakhawy et al. [137], who used ectoine to improve rhizobial survival in liquid inoculants. More directly, nanotechnology provides a robust physical solution. Mehmood et al. [138] reported that nano-carriers buffer inoculants against desiccation and UV stress while enhancing root adherence. Similarly, Aloo et al. [139] concluded that nano-scale materials significantly improve microbial stability under fluctuating environmental conditions. Collectively, these findings affirm that nano-carriers are active protectants that enhance the robustness and field performance of rhizobial inoculants. Silicon nanoparticle matrices, in particular, have demonstrated efficacy in encapsulating rhizobial cells, forming a physical barrier that mitigates environmental stress while preserving microbial viability [118,119].

### 5.3. Rhizosphere Modification and Enhanced Colonization

Beyond physical protection, silicon nanoparticles actively modify the rhizosphere to promote rhizobial colonization. Application of silicon dioxide nanoparticles (SiO_2_ NPs) induces notable changes in legume root architecture, including increased root hair density and elongation. These morphological adaptations expand the root surface area, facilitating rhizobial attachment and enhancing nodule initiation. As root hairs are the primary entry points for rhizobia, their proliferation is closely linked to improved nodulation efficiency.

The importance of root hair morphology is highlighted by Lu et al. [140] in their study of soybean-rhizobia interactions. Recent research suggests that SiO_2_ nanoparticles can also induce beneficial changes in root architecture, as shown by Imtiaz et al. [141] in their work with Ocimum sanctum. These studies support the idea that SiO_2_ nanoparticles act as abiotic elicitors, preparing legume roots for improved microbial interactions. Moreover, silicon-induced alterations in root exudation, including increased secretion of specific flavonoids and organic acids, create a chemically favorable environment for rhizobial growth and chemotaxis [142].

Silicon (Si) plays a multifaceted role in reinforcing plant cell walls, alleviating oxidative stress, and enhancing symbiotic efficiency in legumes. During the infection process, Si deposition in cell walls strengthens root tissue structure, providing a stable scaffold for infection thread development while still permitting the localized remodeling necessary for rhizobial entry. This balance is particularly important in faba beans, where the primary cell wall of root hairs is composed mainly of cellulose, hemicellulose, and pectins, allowing rapid tip growth and maintaining permeability. Root hairs serve as the critical entry point for *Rhizobium* bacteria, and their flexibility enables the curling process around the bacteria—a step that would be impossible if the cell wall were rigidified by lignin. While root hairs themselves are not lignified, deeper vascular tissues such as the xylem are heavily lignified to transport water under pressure, and in older roots undergoing secondary growth, the epidermis may be replaced by a lignified periderm once root hairs have withered away.

Silicon-induced modifications—including moderate lignification, callose accumulation, and elevated antioxidant activity—collectively establish optimal conditions for infection thread stability and nodule formation under stress [29,72,143]. Beyond these localized effects, Si transport and deposition in the epidermis and vascular cortex hardens cell walls to shield plants from external abiotic stresses [144], while Si polymerization improves cell turgidity and wall extension, enhancing transpiration and stomatal regulation. Si also activates antioxidant enzymes that scavenge reactive oxygen species (ROS) generated under salinity stress [145]. Within nodules, Si promotes cell wall thickening and reduces peri-bacteroid space size, facilitating solute transport and accelerating O_2_ diffusion [58,146]. Furthermore, Si decreases the plant’s reliance on lignin, a carbon-intensive compound, thereby allowing more carbon to be allocated to bacteroid respiration and nodule organogenesis, ultimately improving N_2_-fixation efficiency [146,147]. Recent studies confirm that SiO_2_ nanoparticles (n-SiO_2_) significantly enhance nodule health and nitrogen fixation potential [83,84], while co-application of silicon and rhizobia improves nodulation efficiency by synchronizing microbial colonization with enhanced host receptivity [148]. Collectively, these findings underscore the dual role of Si and n-SiO_2_ in strengthening plant cell walls, maintaining root hair flexibility for rhizobial infection, mitigating oxidative stress, and promoting stable symbiotic relationships. This integrated action supports nodule health, nitrogen metabolism, and crop productivity under future climate scenarios characterized by elevated CO_2_.

### 5.4. Synchronized Delivery and Synergistic Effects

A key advantage of nano-biofertilizer formulations is the simultaneous delivery of rhizobia and silicon. This coordinated application ensures the plant receives both the microbial inoculant and the mineral stimulant at the same developmental stage, maximizing synergistic effects. Studies have shown that such co-delivery systems can increase nodulation efficiency by 25–35% compared to the sequential application of individual components [149].

The gradual release of silicic acid from SiO_2_ nanoparticles ensures sustained silicon availability during the critical phases of nodule development and function [97]. At the same time, the controlled release of rhizobia from nano-carriers provides optimal timing for root colonization [148]. Together, this integrated strategy represents a significant advancement over conventional methods, as it optimizes the spatial and temporal coordination essential for successful symbiosis establishment.

The dissolution of SiO_2_ nanoparticles into bioavailable silicic acid (Si(OH)_4_) occurs as a slow and sustained process. Laboratory studies have demonstrated that amorphous silica nanoparticles (20–50 nm) release approximately 0.05–0.25 mg Si/day per gram of particles in aqueous media, with the rate strongly influenced by pH, ionic strength, and particle morphology [150]. This release is not instantaneous but follows a gradual pattern, whereby smaller particles with higher surface area dissolve more rapidly, while larger particles provide a longer-lasting supply.

In soil and plant systems, this dissolution typically continues for 2–4 weeks, ensuring that silicon remains available during the early stages of root colonization, infection thread formation, and nodule organogenesis [151]. Such sustained release contrasts sharply with conventional bulk silica sources, which often leach quickly and lose bioavailability. Importantly, the prolonged release of silicic acid from SiO_2_ nanoparticles aligns with the temporal requirements of rhizobial infection and nodule development, thereby supporting symbiotic efficiency under stress conditions.

### 5.5. Effects on Rhizobial Metabolism

Recent studies confirm that SiO_2_ nanoparticles (Si-NPs) directly influence rhizobial metabolism by modulating gene expression and signaling pathways. Sub-lethal concentrations of Si-NPs have been shown to upregulate nod gene expression, thereby amplifying molecular signaling required for infection thread formation and nodule initiation [94]. This aligns with recent transcriptomic evidence demonstrating that SiO_2_ NPs enhance stress-related and symbiosis-related gene networks, thereby improving microbial compatibility with host plants [152]. Moreover, integrated physiological and metabolomic analyses reveal that SiO_2_ NPs strengthen microbial resilience under abiotic stress, indirectly supporting symbiotic efficiency [153]. These findings suggest that nano-silicon functions not only as a soil amendment but also as a biochemical modulator of rhizobial metabolism, reinforcing early symbiotic signaling and contributing to higher nodule formation rates.

**Figure 10:**
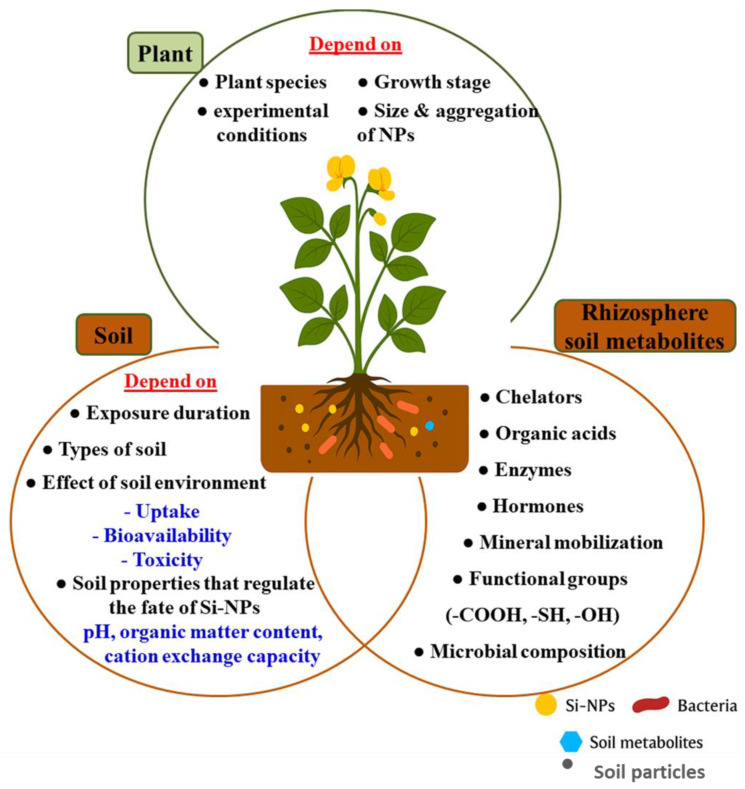
Interactions of silicon nanoparticles (Si-NPs) within the soil–plant–microbe system. This figure summarizes the complex pathways through which Si-NPs influence symbiotic performance and ecosystem dynamics. In soils, Si-NPs improve structure, porosity, and moisture retention, while soil properties (e.g., pH, organic matter) regulate their aggregation, dissolution, and transport. Within the rhizosphere, microbial metabolites such as organic acids and enzymes transform Si-NPs, modulating their solubility, reactivity, and uptake. At the plant interface, smaller and well-dispersed nanoparticles are absorbed more efficiently, enhancing physiological processes including photosynthesis, antioxidant activity, and nutrient transport under stress. Together, these soil, rhizosphere, and plant-level interactions converge to strengthen rhizobial metabolism, upregulate *nod* gene expression, and improve nodule initiation, ultimately enhancing nitrogen fixation efficiency and legume productivity.

The complex interactions of Si-NPs within the soil–plant–microbe system are summarized in Figure 10. This integrative framework highlights three major domains—soils, rhizosphere metabolites, and plants—showing how Si-NPs simultaneously improve soil structure and fertility, regulate microbial metabolism, and enhance plant physiological responses. By explicitly linking the direct effects on rhizobial metabolism (Section 5.5) with the broader ecological impacts on soil environment (Section 5.6), Figure 8 provides a unified perspective on how nano-silicon contributes to enhanced symbiotic efficiency and legume productivity.

### 5.6. Effects on Soil Environment

Nano-silicon amendments also exert ecological effects on soil systems, improving structure, porosity, and moisture retention. This creates favorable physical conditions for plant growth and microbial activity. Recent work highlights that silicon enhances soil microbiome resilience against drought, salinity, and heavy metal stress, thereby stabilizing microbial diversity and fostering rhizobial establishment [154].

Si-NPs interact dynamically with soil properties such as pH and organic matter, which regulate their aggregation, dissolution, and transport [94]. Excessive concentrations, however, may cause toxicity, altering microbial community composition and reducing beneficial taxa. Within the rhizosphere, microbial metabolites (e.g., organic acids, enzymes) play a pivotal role in Si-NP transformation, influencing solubility, reactivity, and uptake [94].

At the plant interface, smaller and well-dispersed Si-NPs are absorbed more efficiently, enhancing physiological processes such as photosynthesis, antioxidant activity, and nutrient transport under stress conditions [155]. Furthermore, Si-NPs modulate gene expression related to stress resistance and nutrient uptake, contributing to improved plant resilience and productivity [156].

## 6. Contribution to Climate Change Mitigation and Adaptation

### 6.1. Climate Adaptation Through Enhanced Stress Resilience

**Figure 11:**
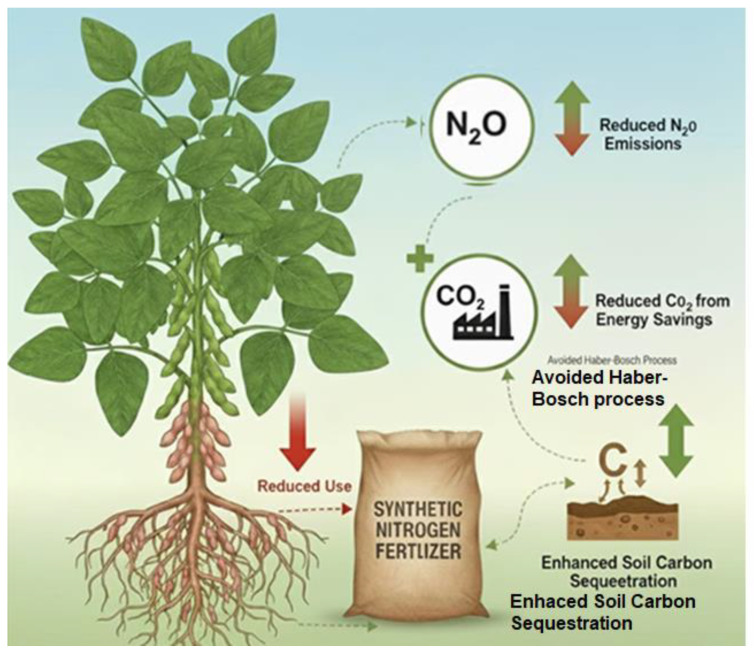
Silicon-enhanced nitrogen fixation and climate benefits. This figure illustrates how silicon supplementation strengthens legume–rhizobium symbiosis, enhancing nod gene expression, infection thread formation, and nodule development. Improved nitrogen fixation efficiency reduces dependence on synthetic nitrogen fertilizers, thereby lowering nitrous oxide (N_2_O) emissions, decreasing carbon dioxide (CO_2_) release from energy-intensive fertilizer production and promoting soil carbon sequestration. Collectively, these benefits highlight the role of silicon in advancing climate-smart agriculture and ecosystem sustainability.

Silicon-mediated enhancement of biological nitrogen fixation (BNF) offers a vital strategy for climate adaptation in the face of increasing environmental variability. As illustrated in Figure 11, by strengthening the legume–rhizobia symbiosis under abiotic stress, silicon directly bolsters agricultural climate resilience. For instance, silicon application can sustain nitrogen fixation rates under drought conditions that would otherwise reduce nodule activity by 40–60% [103].

This capacity to maintain BNF supports legume productivity in water-limited environments, which are becoming more prevalent due to shifting precipitation patterns. The underlying mechanisms involve improved water-use efficiency, preserved nodule function, and sustained rhizobial symbiosis under moisture deficit [148].

Similarly, under salinity stress, silicon mitigates ion toxicity and osmotic imbalance, preserving membrane integrity and enzymatic activity within nodules. This protective effect helps prevent the 50–70% yield losses that are frequently reported in salt-affected legumes [157]. Co-application of silicon with beneficial microbes has proven particularly effective in maintaining nitrogen fixation and overall legume performance under saline conditions [158].

The protective mechanisms underpinning this resilience include enhanced antioxidant defenses and improved osmotic regulation, which collectively shield the nitrogenase complex from stress-induced damage. Furthermore, silicon-treated legumes exhibit superior recovery capacity following drought or heat stress, enabling a rapid restoration of nitrogen fixation. This functional resilience not only safeguards crop yield but also supports long-term soil health by ensuring continuous inputs of organic matter and nitrogen, even under suboptimal growing conditions.

### 6.2. Direct Climate Mitigation Through Greenhouse Gas Reduction

Enhancing BNF through silicon application offers direct mitigation of greenhouse gas (GHG) emissions by reducing dependence on synthetic nitrogen fertilizers. The production and use of these fertilizers account for approximately 2.4–2.5% of global anthropogenic GHG emissions, primarily due to energy-intensive manufacturing and the release of nitrous oxide (N_2_O) during application [159]. N_2_O is an especially potent GHG, with a global warming potential 273–298 times greater than CO_2_ over a 100-year period and an atmospheric lifetime of about 114 years [160].

Silicon-enhanced legume systems show strong potential to reduce fertilizer inputs while maintaining yields. Liang et al. [88] reported that soil treatment with biogeochemically active Si substances allows for a reduction in traditional mineral fertilizer application by 10–40% (some specific tropical cereal-legume contexts show potential for even higher reductions, up to 50%) without compromising yield. El Moukhtari et al. [104] utilized hydroponic and controlled growth chamber environments to examine legumes such as alfalfa (*Medicago sativa*) and faba bean (*Vicia faba*) under salt and drought stress. Their findings indicate that Si application significantly increases the activity of nitrogen-assimilating enzymes, such as nitrate reductase, thereby enhancing nitrogen uptake efficiency; this effectively reduces the pool of residual soil nitrogen available for microbial conversion into N_2_O emissions. Complementing these findings, the meta-analysis by Coskun et al. [161], which synthesized data from both greenhouse and field-scale trials across a broad spectrum of cereals and legumes, provides a mechanistic basis for the projected 30–50% reduction in fertilizer requirements. They highlight that Si simultaneously improves phosphorus (P) availability and nitrogen (N) uptake. In legume systems, this increased P availability is particularly critical, as it provides the necessary energetic substrate to fuel the ATP-dependent process of biological nitrogen fixation within the root nodules.

### 6.3. Indirect Mitigation Through Energy Savings

Silicon treatment contributes to agricultural sustainability through multiple pathways. By enhancing biological nitrogen fixation (BNF) in legumes, silicon reduces reliance on synthetic fertilizers, yielding substantial indirect climate mitigation benefits via energy savings [162]. The Haber–Bosch process, responsible for industrial nitrogen fertilizer production, consumes 1–2% of global fossil fuel energy and contributes significantly to CO_2_ emissions [163]. Strengthening the Rhizobium–legume symbiosis with silicon has been identified as a strategic route for lowering fertilizer inputs and associated emissions [162]. Broader studies indicate that legume-based rotations can reduce energy use by 15–25% compared to conventional systems [163], with potential global savings of 0.5–0.8 gigatons of CO_2_-equivalent emissions annually [164,165]. These reductions extend beyond production to include decreased emissions from fertilizer transportation and application [166,167]. In parallel, silicon treatment influences arthropod diversity by reinforcing plant defense mechanisms. Direct effects include enhanced tissue fortification and biochemical resistance that impair herbivore feeding, while indirect effects involve silicon-mediated changes in volatile organic compound emissions that attract natural enemies and reshape arthropod community structure. Such dual pathways reduce pest pressure while promoting ecological interactions that increase predator and parasitoid abundance, thereby contributing to greater arthropod diversity in cropping systems [168].

### 6.4. Co-Benefits: Enhanced Carbon Sequestration

Silicon application in leguminous crops offers significant co-benefits for soil carbon dynamics through improved root development and organic matter stabilization. Silicon-treated legumes exhibit 20–35% increases in root dry matter, which directly contributes to soil organic carbon accumulation via enhanced belowground biomass input. This increased biomass supports microbial activity and facilitates long-term carbon storage.

Srivastava et al. [166] report that silicon fortification improves root architecture and biomass, delivering 15–25% more carbon to soil systems compared to untreated controls, especially under stress conditions. This is attributed to increased root exudation and improved plant resilience, which sustain carbon deposition even during drought or salinity. Furthermore, silicon interacts with organic molecules and mineral surfaces to form stable carbon-silicon complexes [167]. This interaction reduces decomposition rates and can increase the mean residence time of soil organic carbon by 20–40% [169,170], thereby enhancing long-term carbon sequestration and soil structural integrity.

The benefits of silicon in agriculture are significant, as it stimulates nodulation and biological nitrogen fixation (BNF), leading to increased plant productivity. This results in higher levels of nitrogen- and carbon-rich crop residues, which contribute to the buildup of soil organic matter (SOM). This positive feedback loop enhances soil fertility and structure. Research by Putra et al. [78] showed that silicon-enhanced nodulation in Medicago truncatula increased biomass and root exudation, which are essential for SOM accumulation and microbial activity. Kebede [165] also highlights the important role of legumes in improving soil fertility, emphasizing the broader impact of silicon-mediated enhancements on soil carbon dynamics and overall soil health.

## 7. Challenges and Future Perspectives

### 7.1. Knowledge Gaps and Mechanistic Insights

Despite growing evidence of its agronomic benefits, notable gaps persist in our understanding of silicon’s molecular mechanisms. The field must advance from descriptive observations toward mechanistic, causative frameworks. Emerging studies suggest that silicon can modulate phytohormonal pathways, including cytokinin, auxin, and jasmonic acid, thereby influencing nodule initiation and development [104]. However, whether SiO_2_ nanoparticles act primarily as signaling entities or through dissolution into silicic acid remains unclear. Addressing this question requires advanced transcriptomic and proteomic analyses to test specific hypotheses, such as the preferential upregulation of flavonoid biosynthesis genes (e.g., *CHS*, *IFS*) and components of the Common Symbiotic Signaling Pathway (CSSP) in root hairs under stress [155]. Future work employing hormone mutants and biosensor technologies will be critical to disentangle silicon’s role in phytohormonal cross talk and its contribution to enhanced symbiotic nitrogen fixation.

### 7.2. Optimization and Validation Challenges

A critical challenge is the optimization of silicon application protocols across diverse legume–rhizobia systems. A systematic evaluation of different silicon sources—from conventional silicates and stabilized silicic acid to various nanosilicon formulations—is urgently needed across a range of soil types and environmental conditions [155]. Determining optimal application rates, timing, and delivery methods (e.g., soil amendment, seed coating, foliar spray) is essential, given the interspecific variability in silicon uptake and symbiotic requirements. While pot experiments show promise, large-scale, multi-environment field trials are the crucial next step to validate impacts on nodulation, yield, and long-term soil health and microbial resilience under real-world conditions [104].

Field studies have shown that silicon (Si) supplementation can significantly enhance legume performance in agricultural settings by improving soil properties, crop productivity, and stress resilience [171]. In a two-year field experiment on lucerne (*Medicago sativa*), a key forage legume, researchers applied calcium silicate slag at varying rates. The highest rate increased bioavailable soil Si by 181% and raised soil pH from 5.2 to 6.3, creating more favorable conditions for growth [171]. This resulted in crop yield increases of up to 52%, although the effect varied seasonally. Foliar Si concentrations showed a marginal rise, likely diluted by greater shoot biomass, while forage quality remained unchanged. The treatment also shifted arthropod community structures toward higher diversity without introducing toxic metal contamination or reducing invertebrate abundances, highlighting ecological co-benefits.

Another multi-year investigation examined foliar-applied Si-containing fertilizers (Herbagreen and Optysil) in grass-legume mixtures on temporary grasslands, including species such as *Dactylis glomerata*, *Festulolium braunii*, *Trifolium pratense*, and *Medicago x varia*, under variable weather conditions, including drought. Si application minimally affected botanical composition but markedly improved nutritional quality: crude protein content rose (e.g., from 99.1 g/kg DM in controls to 105.2 g/kg DM with Herbagreen), while crude fiber, neutral detergent fiber, and acid detergent fiber levels decreased, enhancing organic matter and dry matter digestibility. These gains were consistent across years, with Herbagreen slightly more effective, underscoring Si’s role in sustaining forage value amid environmental variability [172].

Building on these forage systems, field research on grain legumes reveals similar advantages. For black gram (*Vigna mungo*), Indian trials in a randomized complete block design combined silixol (a bioavailable Si formulation) as granules (20 kg/ha) and foliar spray (1% at 40 days after sowing) with 50% recommended fertilizer plus vermicompost. This approach significantly boosted growth parameters—plant height to 67.5 cm, dry matter production to 1959 kg/ha, leaf area index to 2.24, chlorophyll content to 94.95 SPAD units, and root nodulation to 667 nodules per plant—yielding 849 kg/ha grain, a 61% increase over full fertilizer controls. Soil health also improved, with microbial biomass carbon up 16%, dehydrogenase activity up 59.6%, and microbial populations up 31.8% [173,174].

For common bean (*Phaseolus vulgaris*), Si fertigation proved effective against drought in Brazilian field trials on eutroferric Red Latosol soil. Under water regimes of 80%, 60%, and 40% soil water retention capacity, Si doses (0–12 kg/ha as sodium silicate, split applications), with or without supplementary potassium, enhanced shoot dry matter, nutrient uptake (N, P, K, Ca, Mg, S, Fe, Zn), relative water content, leaf water potential, photosynthetic pigments, net photosynthesis, and stomatal conductance. Silicon (Si) application has been shown to reduce drought-induced damage by decreasing electrolyte leakage, hydrogen peroxide (H_2_O_2_), and malondialdehyde (MDA) accumulation, while boosting antioxidant enzyme activity like superoxide dismutase (SOD). These physiological enhancements help stabilize cellular membranes, reduce oxidative stress, and promote biomass production under drought conditions. Field studies in wheat have confirmed that Si supplementation can lower leaf electrolyte leakage and increase SOD activity, improving drought tolerance [175]. The effectiveness of Si varies with dosage, with recommended rates of 6 kg/ha under normal irrigation, 7 kg/ha under moderate water deficit, and 8 kg/ha under severe deficit. Research on common beans demonstrates that silicon supplementation improves water use efficiency and nutrient uptake, thereby enhancing tolerance to drought conditions. These results underscore silicon’s potential as a practical tool for sustaining crop productivity under water-limited environments, with application strategies adjusted to the severity of stress.

These peer-reviewed field studies across forage (lucerne) and grain legumes confirm Si’s capacity to boost yields (ranging from 11 to 61% depending on species, dose, and conditions), promote root and nodule development, improve nutrient acquisition, water-use efficiency, and stress tolerance, and often enable reduced fertilizer use. Mechanisms involve soil pH optimization, enhanced microbial activity, and physiological resilience, positioning Si as a sustainable amendment for legume systems worldwide. Although much Si research has targeted grasses, emerging evidence highlights its benefits for rhizobia-legume symbiosis, including improved nodulation, nitrogen fixation, and stress tolerance [97,98]. Further multi-year, multi-location trials across diverse agroecosystems could refine application strategies for broader adoption.

### 7.3. Economic Viability and Scaling Considerations

The high cost of silicon applications, particularly engineered nanoparticles, remains a significant barrier to adoption. Current nanosilicon production methods are economically prohibitive for routine farm use. Future research must prioritize cost-effective ‘green synthesis’ approaches, such as valorizing silica-rich agricultural byproducts like rice husks [155]. A comprehensive techno-economic analysis is needed to compare nano-biofertilizers against conventional peat-based inoculants and synthetic fertilizers, accounting for yield gains and environmental benefits. Concurrently, life cycle assessments will be instrumental in evaluating the environmental footprint and true economic feasibility across different farming systems and scales.

### 7.4. Regulatory Frameworks and Safety Considerations

The regulatory landscape for nano-agricultural inputs, including silicon nanoparticles, is fragmented and underdeveloped. There is an urgent need for clear, science-based guidelines governing product testing, approval, and post-market monitoring. Key priorities include rigorous ecotoxicological assessments of impacts on non-target soil organisms, plant tissues, and food safety. Investigations into the long-term fate and transport of nanoparticles—including their persistence, transformation, and potential for bioaccumulation—are essential for establishing safety thresholds [155]. Harmonizing international standards will facilitate global technology transfer while safeguarding environmental and human health.

### 7.5. A Prioritized Roadmap for Research and Implementation

To advance *Rhizobium*–nanosilicon formulations from concept to field-ready technology, a coordinated and interdisciplinary effort is essential, focused on a three-pronged roadmap: first, developing robust protocols for the co-encapsulation of viable *Bradyrhizobium/Rhizobium* cells and SiO_2_ nanoparticles within stable seed-coating matrices to ensure synchronized release and microbial viability; second, executing rigorous multi-location, multi-year field trials in key legume crops to validate impacts on yield, nitrogen fixation, and soil health under abiotic stress; and third, elucidating the molecular basis of Si/NP-induced resilience using mutant legume lines and multi-omics approaches, while conducting parallel ecotoxicological assessments for comprehensive safety profiling. Successful implementation will further require the development of decision-support tools for farmers and hinges on sustained collaboration among agronomists, soil scientists, molecular biologists, and policy experts to translate these innovations into scalable, sustainable solutions.

## 8. Conclusions

This review establishes silicon not merely as a beneficial element but as a pivotal agent in enhancing the resilience and efficiency of the legume–rhizobia symbiosis. Our analysis of the nodulation process—from the initial molecular dialog to the final stage of nitrogen fixation—reveals multiple critical junctures where silicon, and more effectively nanosilicon, provides indispensable support. The mechanisms encompass enhanced symbiotic signaling, structural reinforcement of infection pathways, mitigation of oxidative and nitrosative stress, and crucial metabolic support for the energy-intensive process of nitrogen fixation.

The emergence of nanosilicon technology represents a paradigm shift, offering superior bioavailability, controlled release properties, and an enhanced capacity to elicit plant defense responses compared to conventional fertilizers. The development of integrated Rhizobium–nanosilicon formulations, or nano-biofertilizers, is particularly promising, as they simultaneously protect microbial inoculants, promote root colonization, and enable synchronized delivery of both biological and mineral components.

From a climate perspective, silicon-enhanced legume systems offer a powerful triple benefit: adaptation through improved abiotic stress resilience, direct mitigation via reduced N_2_O emissions from decreased synthetic fertilizer use, and co-benefits through enhanced soil carbon sequestration. The significant energy savings from reduced reliance on the Haber–Bosch process further amplify this mitigation potential.

Future directions must now address several unresolved questions. The precise molecular mechanisms by which Si-NPs regulate rhizobial gene expression and plant signaling remain incompletely understood. Standardized application protocols are lacking, with dosage, particle size, and delivery methods requiring optimization across diverse agroecosystems. Large-scale field trials are urgently needed to validate laboratory findings under real-world conditions. Cost-effective and sustainable nanosilicon production methods must be developed to ensure scalability. Finally, robust regulatory frameworks and risk assessments are essential to balance the benefits of Si-NPs with potential ecological and health concerns.

The strategic integration of silicon and nanosilicon technologies into legume production systems offers a transformative pathway toward a more productive, efficient, and climate-resilient agriculture. Realizing this vision demands interdisciplinary collaboration—spanning plant biology, nanotechnology, soil science, climate science, and policy—and sustained investment. The potential payoff is immense: a significant reduction in agriculture’s environmental footprint alongside enhanced global food security.

## 9. Future Research Priorities

Future research on silicon and nano-biofertilizers should focus on elucidating the molecular mechanisms underlying their role in enhancing legume–rhizobium symbiosis. Omics-based approaches, including transcriptomics, proteomics, and metabolomics, are needed to clarify how silicon regulates nod gene expression, antioxidant defense, and leghemoglobin stabilization. At the technological level, optimization of nano-carriers such as SiO_2_, chitosan, and polymeric nanoparticles remains a priority, with emphasis on particle size, dosage, and surface modifications to ensure safe and efficient microbial delivery. Co-delivery systems that combine rhizobia with signaling molecules, such as flavonoids, represent a promising avenue for improving nodulation efficiency. Equally important are long-term ecotoxicological studies to assess nanoparticle persistence, interactions with soil microbiomes, and potential environmental risks under diverse agroecological conditions. Transitioning from controlled experiments to multi-location field trials will be essential to validate efficacy, scalability, and farmer adoption. In parallel, research should quantify the contributions of nano-biofertilizers to climate change mitigation, including reduced greenhouse gas emissions, enhanced soil carbon sequestration, and improved plant resilience under stress. Finally, the establishment of clear policy and regulatory frameworks will be critical to guide safe deployment, address biosafety concerns, and ensure cost-effectiveness. Collectively, these priorities define a roadmap for advancing nano-biofertilizer technology as a cornerstone of sustainable, climate-smart agriculture.

## Figures and Tables

**Table 1:** Examples of flavonoids and their role in nod factor activation.

Flavonoid Compound	Legume Source	Rhizobial Target	Activated Nod Factor/Gene	References
Luteolin (C_15_H_10_O_6_) 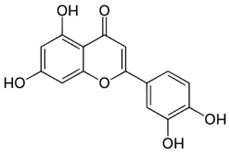 3′,4′,5,7-Tetrahydroxyflavone	Alfalfa (*Medicago sativa*)	*Sinorhizobium meliloti*	Activates nodD1, leading to synthesis of lipochitooligosaccharide Nod factors	[10]
Daidzein (C_15_H_10_O_4_) 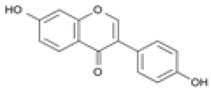 4′,7-Dihydroxyisoflavone	Soybean (*Glycine max*)	*Bradyrhizobium japonicum*	Induces nodYABCSUIJ operon via NodD	[11]
Genistein (C_15_H_10_O_5_) 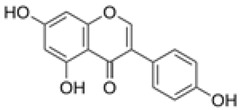 4′,5,7-Trihydroxyisoflavone	Soybean (*Glycine max*)	*Bradyrhizobium japonicum*	Activates nodD1 and downstream nod genes	[11]
Eriodictyol (C_15_H_12_O_6_) 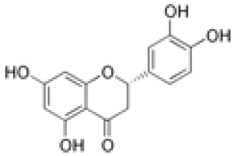 (2*S*)-3′,4′,5,7-Tetrahydroxyflavan-4-one	Pea (*Pisum sativum*)	*Rhizobium leguminosarum*	Stimulates nodD-dependent transcription	[14]
Hesperetin (C_16_H_14_O_6_) 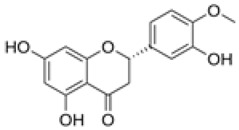 (2*S*)-3′,5,7-Trihydroxy-4′-methoxyflavan-4-one	fenugreek (*Trigonella foenum graecum*) symbi	*R. tibeticum*	Alleviating the inhibitory effect of heavy metal stresses on *nod* gene expression	[15,16]
Apigenin(C_15_H_10_O_5_) 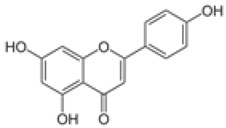 4′,5,7-Trihydroxyflavone	fenugreek (*Trigonella foenum graecum*) symbi	*R. tibeticum*	Alleviating the inhibitory effect of salinity stresses on *nod* gene expression	[17]
Naringenin (C_15_H_12_O_5_) 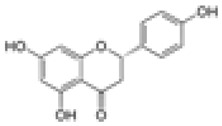 (2*S*)-4′,5,7-Trihydroxyflavan-4-one	Common bean (*Phaseolus vulgaris*)	*Rhizobium etli*	Induces nodA, nodB, nodC via NodD	[18]
Liquiritigenin (C_15_H_12_O_4_) 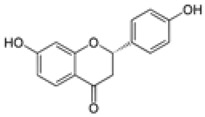 (2*S*)-4′,7-Dihydroxyflavan-4-one	Lotus (*Lotus japonicus*)	*Mesorhizobium loti*	Activates nodD and Nod factor biosynthesis genes	[18]

**Table 2:** Stage-specific sensitivity of legume nodulation to drought and salinity stress.

Stage of Symbiosis	Drought Sensitivity	Salinity Sensitivity	Quantitative Thresholds	Molecular Mechanisms of Disruption	References
Flavonoid signaling and rhizobial recognition	Highly sensitive—reduced flavonoid exudation and root hair growth	Moderately sensitive—altered flavonoid profiles and reduced Nod factor perception	Drought: soil water potential < −0.5 MPa reduces flavonoid release; Salinity: >50 mM NaCl alters flavonoid/NodD interactions	Drought suppresses phenylpropanoid pathway enzymes; salinity interferes with Nod factor binding to LysM receptors	[45]
Infection thread initiation and progression	Sensitive—reduced root hair curling and IT elongation	Very sensitive—IT wall integrity disrupted, rhizobial movement impaired	Drought: >30% reduction in IT frequency under water deficit; Salinity: >75 mM NaCl causes IT collapse	Drought reduces cytoskeletal rearrangements; salinity alters exopolysaccharide (EPS) production and pectin remodeling	[46]
Nodule organogenesis	Sensitive—suppressed cortical cell division and primordium formation	Moderately sensitive—delayed primordium development	Drought: −0.8 MPa reduces cortical cell division by >40%; Salinity: >100 mM NaCl delays primordium initiation	Drought downregulates cytokinin signaling; salinity interferes with auxin redistribution	[46]
Nitrogen fixation (nitrogenase activity)	Very sensitive—reduced ATP supply and leghemoglobin stability	Extremely sensitive—direct inhibition of nitrogenase, ROS accumulation	Drought: >50% reduction in nitrogenase activity under −1.0 MPa; Salinity: >100 mM NaCl reduces nitrogenase activity by 60–70%	Drought reduces photosynthate supply and leghemoglobin stability; salinity induces ionic toxicity (Na^+^, Cl^−^), ROS and nitric oxide inhibition of nitrogenase	[45,46]

**Table 3:** Effects of silicon and nanosilicon on legume symbiotic performance.

Parameter	Observed Effect	Legume Species	Proposed Mechanism	References
Nodulation	Nodule number ↑ 48.3%; biomass ↑ 53.6%	Soybean	Upregulation of chalcone synthase (CHS) genes and root hair integrity under elevated CO_2_	[102]
Nitrogen Metabolism	N-assimilation enzymes ↑ 38.5–52.1%	Soybean	Improved N-metabolism and assimilation	[9]
Antioxidant Defense	Antioxidant enzyme activity ↑ 15–22%; MDA and H_2_O_2_ ↓ 18–22%	Soybean	Upregulation of SOD, CAT, APX under drought stress	[82,83]
Stress Resilience	Sustained nitrogenase activity under drought/salinity	Lentil (model legume)	Combined structural, osmotic, and oxidative protection	[103]
Symbiotic Signaling	Promoted root hair development; stimulated flavonoid exudation	Soybean	Enhanced flavonoid profile for improved nod gene activation and partner recognition	[104]
Structural and Metabolic Support	Strengthened root hairs and infection threads; improved photosynthetic efficiency	Model legumes	Silica polymerization in cell walls; protection of chloroplast ultrastructure	[104,105]

Key: ↑ = Increase; ↓ = Decrease; MDA = Malondialdehyde; H_2_O_2_ = Hydrogen peroxide; SOD = Superoxide Dismutase; CAT = Catalase; APX = Ascorbate Peroxidase.

**Table 5:** Comparison of nano-carrier materials for rhizobial formulations.

Nano-Carrier	Key Features	Benefits for Rhizobial Delivery	Limitations
SiO_2_ NPs	High stability, stress tolerance	Stimulates flavonoid exudation, nod gene activation, stress resilience	Potential ecotoxicity, dosage optimization needed
Chitosan NPs	Biodegradable, antimicrobial	Protects rhizobia, controlled release, pathogen suppression	Sensitive to pH, may require crosslinking
Polymeric NPs	Tunable degradation, versatile encapsulation	Sustained microbial release, co-delivery of signals/nutrients	Higher production cost, scalability challenges

## Data Availability

The original contributions presented in this study are included in the article. Further inquiries can be directed to the corresponding author.
